# Growth hormone-releasing hormone antagonist MIA-602 inhibits inflammation induced by SARS-CoV-2 spike protein and bacterial lipopolysaccharide synergism in macrophages and human peripheral blood mononuclear cells

**DOI:** 10.3389/fimmu.2023.1231363

**Published:** 2023-08-15

**Authors:** Giuseppina Granato, Iacopo Gesmundo, Francesca Pedrolli, Ramesh Kasarla, Laura Begani, Dana Banfi, Stefania Bruno, Tatiana Lopatina, Maria Felice Brizzi, Renzhi Cai, Wei Sha, Ezio Ghigo, Andrew V. Schally, Riccarda Granata

**Affiliations:** ^1^ Department of Medical Sciences, Division of Endocrinology, Diabetes and Metabolism, University of Turin, Turin, Italy; ^2^ Department of Medical Sciences, University of Turin, Turin, Italy; ^3^ Molecular Biotechnology Center, University of Turin, Turin, Italy; ^4^ Endocrine, Polypeptide, and Cancer Institute, Veterans Affairs Medical Center, Miami, FL, United States; ^5^ South Florida VA Foundation for Research and Education, Veterans Affairs Medical Center, Miami, FL, United States; ^6^ Department of Medicine, Divisions of Medical/Oncology and Endocrinology, and the Department of Pathology, Miller School of Medicine, University of Miami, Miami, FL, United States; ^7^ Sylvester Comprehensive Cancer Center, Miller School of Medicine, University of Miami, Miami, FL, United States

**Keywords:** GHRH, GHRH antagonist, SARS-CoV-2 spike protein, macrophages, peripheral blood mononuclear cells (PBMC), inflammation

## Abstract

COVID-19 is characterized by an excessive inflammatory response and macrophage hyperactivation, leading, in severe cases, to alveolar epithelial injury and acute respiratory distress syndrome. Recent studies have reported that SARS-CoV-2 spike (S) protein interacts with bacterial lipopolysaccharide (LPS) to boost inflammatory responses *in vitro*, in macrophages and peripheral blood mononuclear cells (PBMCs), and *in vivo*. The hypothalamic hormone growth hormone-releasing hormone (GHRH), in addition to promoting pituitary GH release, exerts many peripheral functions, acting as a growth factor in both malignant and non-malignant cells. GHRH antagonists, in turn, display potent antitumor effects and antinflammatory activities in different cell types, including lung and endothelial cells. However, to date, the antinflammatory role of GHRH antagonists in COVID-19 remains unexplored. Here, we examined the ability of GHRH antagonist MIA-602 to reduce inflammation in human THP-1-derived macrophages and PBMCs stimulated with S protein and LPS combination. Western blot and immunofluorescence analysis revealed the presence of GHRH receptor and its splice variant SV1 in both THP-1 cells and PBMCs. Exposure of THP-1 cells to S protein and LPS combination increased the mRNA levels and protein secretion of TNF-α and IL-1β, as well as IL-8 and MCP-1 gene expression, an effect hampered by MIA-602. Similarly, MIA-602 hindered TNF-α and IL-1β secretion in PBMCs and reduced MCP-1 mRNA levels. Mechanistically, MIA-602 blunted the S protein and LPS-induced activation of inflammatory pathways in THP-1 cells, such as NF-κB, STAT3, MAPK ERK1/2 and JNK. MIA-602 also attenuated oxidative stress in PBMCs, by decreasing ROS production, iNOS and COX-2 protein levels, and MMP9 activity. Finally, MIA-602 prevented the effect of S protein and LPS synergism on NF-кB nuclear translocation and activity. Overall, these findings demonstrate a novel antinflammatory role for GHRH antagonists of MIA class and suggest their potential development for the treatment of inflammatory diseases, such as COVID-19 and related comorbidities.

## 1 Introduction

To date, the cases of coronavirus disease 2019 (COVID-19) caused by severe acute respiratory syndrome coronavirus 2 (SARS-CoV-2) have exceeded 758 million, with 6.8 million deaths worldwide (1). The most common symptoms of COVID-19 are fever, cough, dyspnea, and myalgia, while severe disease encompasses pulmonary and systemic hyperinflammation, leading to sepsis, multiorgan failure and acute respiratory distress syndrome (ARDS) (2, 3). SARS-CoV-2 is made up of four structural proteins, of which the spike (S) protein is responsible for cell entry by binding to angiotensin-converting enzyme 2 (ACE2) (4). Receptor engagement leads to virus entry in host cell, by either endocytosis and subsequent S2 subunit cleavage by cathepsins, or activation and cleavage of S2 at the cell surface by transmembrane protease, serine 2 (TMPRSS2) (5). Viral components and products of apoptotic and necrotic cells promote inflammatory responses in innate immune cells such as macrophages, monocytes, and neutrophils, *via* pattern recognition receptors (PRRs) (6). Subsequently, PRRs, including toll-like receptors (TLRs), NOD-like receptors (NLRs) and RIG-I-like receptors (RLRs), induce activation of transcription factors like nuclear factor kappa-light-chain-enhancer of activated B cells (NF-κB), leading to production of inflammatory cytokines/chemokines, cytokine storm and lung inflammation. Indeed, COVID patients show high levels of interleukin (IL)-1, IL-6, IL-8, tumor necrosis factor alpha (TNF-α), and monocyte chemoattractant protein (MCP)-1, among others (2, 3). In addition, SARS-CoV-2 triggers an excess of reactive oxygen species (ROS) and enhances oxidative stress, resulting in more severe disease (7).

Recent studies have demonstrated that S protein interacts with low levels of bacterial lipopolysaccharide (LPS), a known TLR4 ligand, to boost NF-κB activation and inflammatory cytokine release *in vitro*, in THP-1-derived macrophages and human peripheral blood mononuclear cells (PBMCs), and *in vivo* (8–10). Moreover, elevated levels of LPS have been observed in severe COVID-19, often associated with increased death of hospitalized patients (11, 12). Thus, identifying drugs that counteract oxidative stress and inflammation, also induced by S protein and LPS synergism, would help define novel therapeutic approaches to ameliorate hyperinflammation during COVID-19.

Growth hormone-releasing hormone (GHRH) is a hypothalamic hormone that stimulates the synthesis and release of GH from the anterior pituitary (13). In addition, several studies have demonstrated that GHRH and its agonistic analogs exert many peripheral activities, promoting cell growth in both nonmalignant and tumor cells and displaying protective effects in different cells and tissues, through binding to GHRH receptors (GHRH-Rs) (14–18). Moreover, an autocrine/paracrine stimulatory loop formed by locally produced GHRH and its receptors, particularly the splice variant 1 (SV1), promotes the growth of many cancers, a mechanism that can be blocked by GHRH antagonists. These molecules can also inhibit the autocrine/paracrine proliferative effects of tumoral insulin-like growth factor (IGF)-I and -II (19). In the past decades, many GHRH antagonists have been developed and tested in our laboratories, including the latest of Miami (MIA) and AVR class (19–22).

The potent anticancer effects of MIA antagonists have been demonstrated in several *in vitro* and *in vivo* models of human cancer, including lung and colorectal cancer, mesothelioma and pituitary tumors (22–27). These compounds, characterized by increased receptor binding affinity, also exhibited strong antinflammatory and antioxidant activities, in both malignant and non-malignant models, while displaying only weak endocrine GH inhibitory effects (22, 25, 28, 29). In fact, MIA-602 and MIA-690 alleviated LPS-induced ocular inflammation (30, 31), reduced prostatic enlargement and inflammation (32), blunted colon and lung inflammation (33, 34), inhibited inflammation in a model of sarcoidosis (35), and supported lung endothelial barrier function (36). Mechanistically, these effects included attenuation of lymphocyte and macrophage recruitment, inhibition of cytokine release and inflammatory pathways, such as MAPKs, JAK2/STAT3, NF-кB, as well as attenuation of oxidative markers, including ROS, cyclooxygenase (COX)−2, and inducible nitric oxide synthase (iNOS) (27, 28, 30, 32, 34, 36, 37). Overall, the above-mentioned studies suggest potential protective effects of GHRH antagonists against systemic hyperinflammation and sepsis-induced ARDS, main characteristics of severe COVID-19 (2); however, the role of these peptides in SARS-CoV-2-induced inflammation remains unexplored. Thus, based on the foregoing, we aimed to verify whether MIA-602 reduces inflammation in human THP-1-derived macrophages and PBMCs exposed to SARS-CoV-2 S protein and LPS synergism. Additionally, we examined the signaling pathways involved in the effects of the peptide.

## 2 Materials and methods

### 2.1 Reagents

GHRH-R antagonists MIA602 was prepared by Dr. Renzi Cai and Dr. Andrew Schally at the Veterans Affairs Medical Center, University of Miami, Miami, FL, as described previously (25). The chemical structure of MIA-602 is ([(PhAc-Ada)^0^-Tyr^1^, D-Arg^2^, Fpa_5_^6^, Ala^8^, Har^9^, Tyr(Me)^10^, His^11^, Orn^12^, Abu^15^, His^20^,Orn^21^, Nle^27^, D-Arg^28^, Har^29^]hGH-RH(1-29)NH_2_). MIA-602 was dissolved in 100% dimethyl sulfoxide (DMSO) (Merk Life Science, Billerica, MA, USA) and diluted with appropriate incubation medium. The concentration of DMSO never exceed 0.1% (vol/vol). Trypsin, lipopolysaccharides (LPS) from Escherichia coli serotype O111:B4, bovine serum albumin (BSA), 4’,6-diamidino- 2-phenylindole (DAPI), formaldehyde solution, Triton X-100, Bicinchoninic Acid (BCA) protein assay kit, all primers for Real-Time PCR and Coomassie Brilliant Blue R-250 were obtained from (MilliporeSigma, Milan, Italy). RT-PCR, Real-Time PCR reagents and TaqMan probes were obtained from Life Technologies (Thermo Fisher Scientific, Milan, Italy). PepTivator SARS-CoV-2 Spike protein (S protein, code: 130-126-700) was purchased from Miltenyi Biotec (Bologna, Italy). Western blot reagents were purchased from Bio-Rad (Milan, Italy). Rabbit polyclonal antibodies against GHRH-R (code: ab76263) and ERK (code: ab16869) were obtained from Abcam (Cambridge, UK). Rabbit monoclonal primary antibodies against NF*k*B p65 (code: 8242) and P-NF*k*B p65 (code: 3033), glyceraldehyde-3- phosphate dehydrogenase (GAPDH), (code: 2118), P-ERK (code: 3192), SAPK/JNK (code: 9252), P-SAPK/JNK (code: 4668), STAT3 (code:30835) and P-STAT3 (code:9131) were obtained from Cell Signaling Technology (Danvers, MA, USA). Horseradish peroxidase-conjugated goat anti-mouse and goat anti-rabbit-IgG secondary antibodies were from Santa Cruz Biotechnology (Dallas, TX, USA). Goat normal serum and Alexa Fluor-488 goat anti-rabbit secondary antibody were obtained from Jackson Immuno-Research Europe (Newmarket, UK). All other chemicals were purchased from MilliporeSigma (Milan, Italy).

### 2.2 Cell culture and treatments

The THP-1 cells (ATCC, TIB-202) and MSTO-211H human biphasic pleural mesothelioma cell line were purchased from American Type Culture Collection (ATCC; Manassas, VA, USA) and cultured in Roswell Park Memorial Institute (RPMI) 1640 supplemented with 10% fetal bovine serum (FBS), 2-ß-mercaptoethanol (0.05 mM), penicillin (100 U/ml), streptomycin (100 µg/ml) and amphotericin B (250 ng/ml) (MilliporeSigma, Milan, Italy) at 37°C in a 5% CO_2_ humidified atmosphere. Differentiation of THP-1 cells in macrophage-like cells was induced by adding phorbol-12-myristate-13-acetate (PMA) (MilliporeSigma, Milan, Italy) (25 nM) for 48 h, as previously described (38). Differentiated THP-1 cells were maintained in PMA-free medium for further 24 h, then stimulated with S protein (500 ng/ml), MIA-602 (MIA, 1 µM) or LPS (50 ng/ml) alone for 24 h or pretreated with LPS for 3 h then incubated up to 24 h with S protein, in the presence or absence of MIA-602, at the same concentrations. PBMCs were isolated from human peripheral blood of healthy male donors (n=10, age range 30-65 years by Histopaque^®^-1077 density gradient centrifugation (MilliporeSigma) following the manufacturer’s instructions. Cells were maintained in RPMI-1640 with 10% FBS, penicillin (100 U/ml), streptomycin (100 µg/ml) and amphotericin B (250 ng/ml) at 37°C in a 5% CO_2_ humidified atmosphere, then exposed to either LPS (10 ng/ml), S protein (500 ng/ml), MIA-602 (1 µM) alone for 24 h or pre-treated with LPS for 3 h, then for further 24 h with either S protein, MIA-602 or both, at the same concentrations. Untreated cells were used as control.

### 2.3 Western blotting

Protein extraction and Western blot analysis were performed as described previously (15, 25, 39). Briefly, 50 µg proteins were resolved in 10% SDS-PAGE and transferred to a nitrocellulose membrane. After blocking with 1% BSA in Tris-buffered saline with 0.1% Tween for 2 h at room temperature, membranes were incubated overnight at 4°C with the specific antibody (dilution 1:1000). Subsequently, blots were re-probed with the respective total antibodies (1:1000) or GAPDH (1:1500) for normalization. Immunoreactive proteins were visualized using horseradish peroxidase-conjugated goat anti-mouse (1:4000) and goat anti-rabbit (1:10000) secondary antibodies by enhanced chemiluminescence substrate (ECL, Bio-Rad, Milan, Italy) using ChemiDoc XRS (Bio-Rad, Milan, Italy); densitometric analysis was performed with Quantity One software (Bio-Rad, Milan, Italy). Each experiment was performed in triplicate. Densitometric analysis was carried out with Quantity One software (Bio-Rad).

### 2.4 Fluorescence microscopy

THP-1 and MSTO-211H cells were plated on glass coverslips in 35-mm dishes at a density of 5 x 10^5^ cells and then treated with the different stimuli for 24 h. Next, cells were washed once with ice-cold 1X PBS, fixed with 4% paraformaldehyde for 5 min, washed again for three times and stored at 4°C until analysis. PBMCs (3 x 10^5^ cells) were immobilized on glass microscope slides by cytospin and then fixed in 90% ethanol/acetic acid (2:1 solution) at -20°C for 5 min. After fixation, all cells were permeabilized with 0.1% Triton X-100 for 2 min, blocked in goat normal serum (dilution 1:10 in 1X PBS) for 30 min at room temperature and stained overnight at 4°C with rabbit polyclonal anti-GHRH-R (1:200) or anti-NF*k*B (1:200) antibody. The day after, the cells were incubated for 1 h at room temperature with Alexa Fluor 488- conjugated goat anti-rabbit secondary antibody diluted 1:300 in 1X PBS. Then, cells were washed three times and counterstained with DAPI for 15 min at room temperature. Slides were mounted using Fluoromount-G Mounting Medium (MilliporeSigma). Images were captured using a Leica DM200 fluorescent microscope and a Leica DFC340 FX camera (Leica, Solms, Germany) at 40x magnification.

### 2.5 Real-time PCR

Total RNA and reverse transcription to cDNA (1 µg of RNA) from THP-1 cells and PBMCs were performed as described previously (26). RNA purity and quantification were detected spectrophotometrically by 260/280 nm absorbance ratio with NanoDrop One (Thermo Fisher Scientific, Milan, Italy). For real-time PCR, reaction was performed with 50 ng cDNA, 100 nM of each primer, and the Luna Universal qPCR Master Mix (New England BioLabs, Ipswich, MA, USA) using the ABI-Prism 7300 (Applied Biosystems; Thermo Fisher Scientific, Milan, Italy). The following primer pairs [designed with the Primer 3 Software (http://www.primer3.org/)] were used: TNF-α, forward 5'-ATGAGCACTGAAAGCATGATCC-3', reverse 5'-GAGGGCTGATTAGAG AGAGGTC-3' (NM_000594.4); IL-6, forward 5'-AACCTGAACCTTCCAAAGATGG−3', reverse 5'-TCTGGCTTGTTCCTCACTACT-3' (NM_001371096.1); IL-8, forward 5'-TGGACCACACTG CGCCAACAC-3', reverse 5'-ACTTCTCCACAACCCTCTGCA-3' (NM_001354840.3); IL-1β, forward 5'-CCTGTCCTGCGTGTTGAAAGA-3' reverse 5'-GGGAACTGGGCAGACTCAAA-3' (XM_054341810.1); 18S rRNA, forward 5′-CCCATTCGAACGTCTGCCCTATC-3′, reverse 5′-TG CTGCCTTCCTTGGATGTGGTA-3′ (NR_146144.1). 18S rRNA was used as endogenous control. Real-time PCR for MCP1 was performed using commercially available primers [TaqMan Gene Expression Assays; FAM dye labeled TaqMan MGB probe: HS00234140_m1 (MCP1)]. 18S [probe: Hs03003631_g1 (18S)] was used as housekeeping gene. Relative quantification was performed using the comparative Ct (2^-ΔΔCt^) method.

### 2.6 ELISA

THP-1-derived macrophages and PBMCs were seeded in 6-well plates at the concentrations of 6 x 10^6^ cells/well. Conditioned medium from treated cells was collected and centrifuged at 300 x g for 10 min, then stored at -80°C until analysis. TNF-α, IL-1β and IL-6 were measured using human DuoSet ELISA Kit (R&D Systems, Minneapolis, MN, USA), following the manufacturer’s instructions. Absorbance was assessed at 450 nm using the LT-4000 microplate reader (Euroclone, Milan, Italy).

### 2.7 ROS detection

Intracellular ROS levels were measured using the Muse Oxidative Stress Cell Kit (Luminex, Austin, TX, USA) according to the manufacturer’s instructions. Briefly, PBMCs were seeded in 24-well plate (1 x 10^6^ cells/well) and treated with the different stimuli for 24 h. Collected cells were resuspended in Muse Oxidative Stress reagent, incubated at 37°C for 30 min and analysis was performed using Muse Cell Analyzer and Muse Cell Analyzer Software (Luminex, Austin, TX, USA).

### 2.8 Gelatin zymography

Gelatinolytic activity of MMP9 in PBMC conditioned medium was determined by gelatin zymography. Equal amounts of proteins (30 μg) were separated in SDS-PAGE under non reducing conditions without boiling on 10% SDS-polyacrylamide gel co-polymerized with 2 mg/ml of gelatin. After electrophoresis (35 mA for 150-210 min at 4°C), gel was washed in 2.5% Triton X-100 solution for 1 h to remove SDS and then incubated in developing buffer (50 mM Tris-HCl, pH 7.5, 200 mM NaCl, 5 mM CaCl_2_ and 5 µM ZnCl_2_) at 37°C for 48 h, which allows substrate degradation. Finally, gel was fixed in 30% methanol and 10% acetic acid for 30 min, stained with 0.1% Coomassie Brilliant Blue R-250 and destained in 50% methanol and 5% acetic acid. Proteolytic bands were visualized as clear band against a blue background. MMP9 enzymatic activity was determined by quantification of clear bands with ImageJ software (http://rsbweb.nih.gov/ij/).

### 2.9 Statistical analysis

Results are presented as mean ± SEM. Significance was calculated by one-way ANOVA followed by Tukey’s multiple comparison test for *post-hoc* analysis. Analysis was performed using GraphPad Prism 8.0 (San Diego, CA, USA). Significance was established at *P <*0.05.

## 3 Results

### 3.1 GHRH-R and SV1 are expressed in THP-1-derived macrophages and human PBMCs

**Figure 1:**
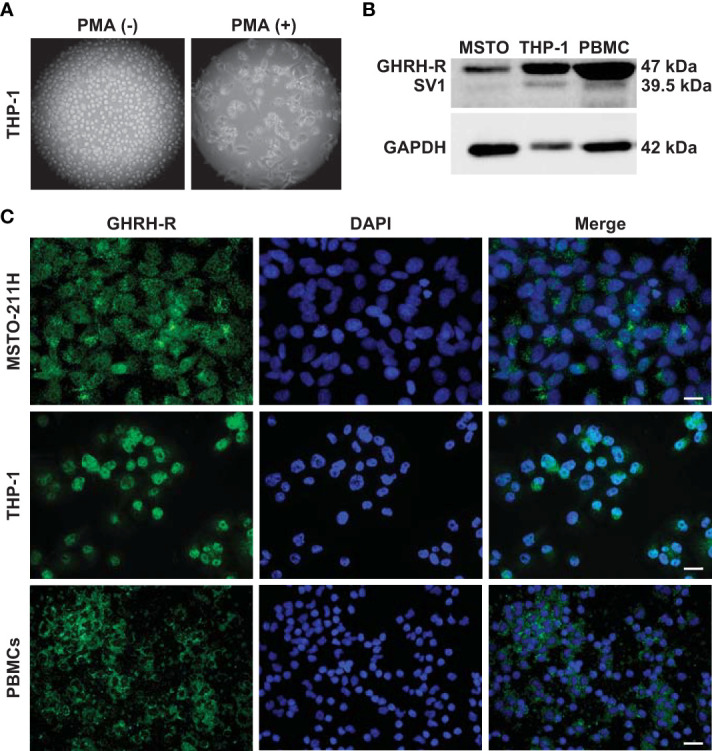
Expression of GHRH-R and SV1 in THP-1-derived macrophages and PBMCs. **(A)** Representative phase contrast microscopy images (magnification, X10) of THP-1 monocytes (left panel) differentiated into macrophage-like cells (right panel) after treatment for 48 h with PMA (25 nM). **(B)** Western blot analysis for GHRH-R and SV1 in THP-1-derived macrophages and PBMCs (top). MSTO-211H pleural mesothelioma cells were used as positive control. Equal protein loading was determined by reprobing with antibody to GAPDH (bottom). **(C)** Representative fluorescence microscopy micrographs showing GHRH-R expression (green) in THP-1-derived macrophages (middle panels) and PBMCs (bottom panels). Nuclei were stained blue with DAPI. MSTO-211H cells were used as positive control (top panels) (scale bar: 200 μm).

THP-1 monocytes were first differentiated into macrophage-like cells. Light microscopy analysis showed that after treatment with PMA, the round shape non-adherent cells became adherent and acquired the typical flat, elongated, and branching macrophage morphology (**Figure 1A**). Because GHRH agonists and antagonists display their biological effects through binding to GHRH-R and/or its splice variant SV1, we assessed the presence of these receptors in THP-1-derived macrophages and human PBMCs. Western blot analysis, using a polyclonal antibody against a common segment of GHRH-R and SV1, showed strong expression of GHRH-R protein and low levels of SV1 in THP-1 cells and PBMCs (**Figure 1B**). Immunofluorescence analysis confirmed the presence of GHRH-R in both cell types (**Figure 1C**). MSTO-211H pleural mesothelioma cells, which express GHRH-Rs (24), were used as positive control in both experiments.

### 3.2 MIA-602 reduces cytokine and chemokine production in THP-1-derived macrophages treated with S protein and LPS combination

**Figure 2:**
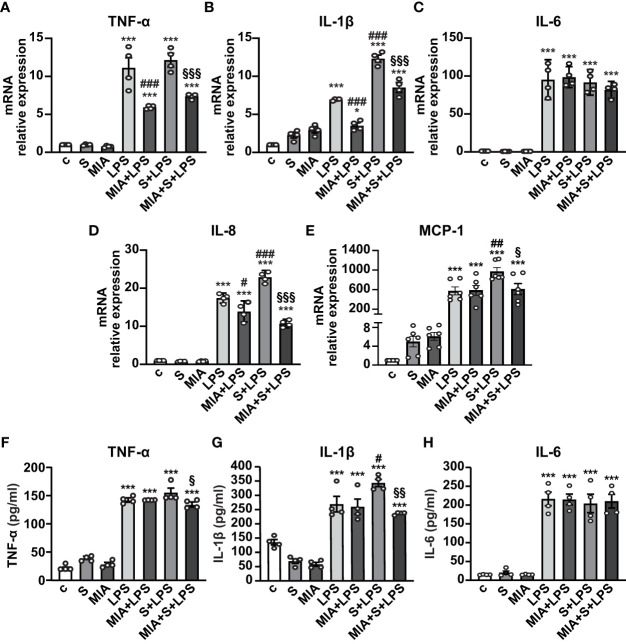
Inhibitory effect of MIA-602 on cytokine and chemokine expression in THP-1-derived macrophages. The cells were either untreated (c, control) or exposed to S protein (S, 500 ng/ml), MIA-602 (MIA, 1 µM) or LPS (50 ng/ml) alone for 24 h or pretreated with LPS for 3 h and then up to 24 h with S protein, in the presence or absence of MIA-602, at the same concentrations. **(A–E)** mRNA levels of TNF-α, IL-1β, IL-6, IL-8 and MCP-1 assessed by real time-PCR and normalized to 18S rRNA. **(F–H)** TNF-α, IL-1β and IL-6 secretion measured by ELISA in cell conditioned medium. Results are means ± SEM. *P < 0.05; ***P < 0.001 vs. control (c); ^#^ *P* < 0.05, ^##^ *P* < 0.01, ^###^ *P* < 0.001 vs. LPS; ^§^ *P* < 0.05, ^§§^ *P* < 0.01, ^§§§^ *P* < 0.001 vs. LPS+S protein by one-way ANOVA and Tukey’s *post-hoc* test (*n* = 4).

To evaluate the antinflammatory role of MIA-602, THP-1 cells were stimulated with S protein, either alone or with LPS. Both S protein and MIA-602 as single agents had no effect on the mRNA levels of TNF-α, IL-6 and IL-8. As expected, the treatment with LPS for 24 h strongly increased TNF-α, IL-1β, IL-6 and IL-8 levels, compared with control, an effect particularly evident for IL-6. The addition of S protein produced a further elevation in IL-1β and IL-8, compared with LPS alone, while TNF-α and IL-6 were not significantly different. MIA-602, used at 1 µM, in agreement with our previous studies (24, 26), blunted the LPS-driven increase of all cytokines, except IL-6. Furthermore, the combination of MIA-602 with S protein and LPS strongly inhibited TNF-α, IL-1β and IL-8 mRNAs, compared with S protein+LPS alone, while IL-6 was slightly, but not significantly, reduced (**Figures 2A-D**). Similar results were obtained for MCP-1 chemokine (also known as C-C motif chemokine ligand 2, CCL2), whose mRNA levels were inhibited by the combination of MIA-602 with S protein and LPS, compared with S protein and LPS alone (**Figure 2E**). In line with the mRNA results, MIA-602 attenuated the production of TNF-α and IL-1β proteins in THP-1 cells exposed to S protein and LPS, while showing no effect on IL-6. (**Figures 2F-H**). Overall, these results indicate the ability of MIA-602 to reduce inflammation in THP-1-derived macrophages, particularly under S protein and LPS synergism.

### 3.3 MIA-602 inhibits NF-κB, JAK-STAT and MAPK inflammatory pathways in THP-1-derived macrophages treated with S protein and LPS combination

**Figure 3:**
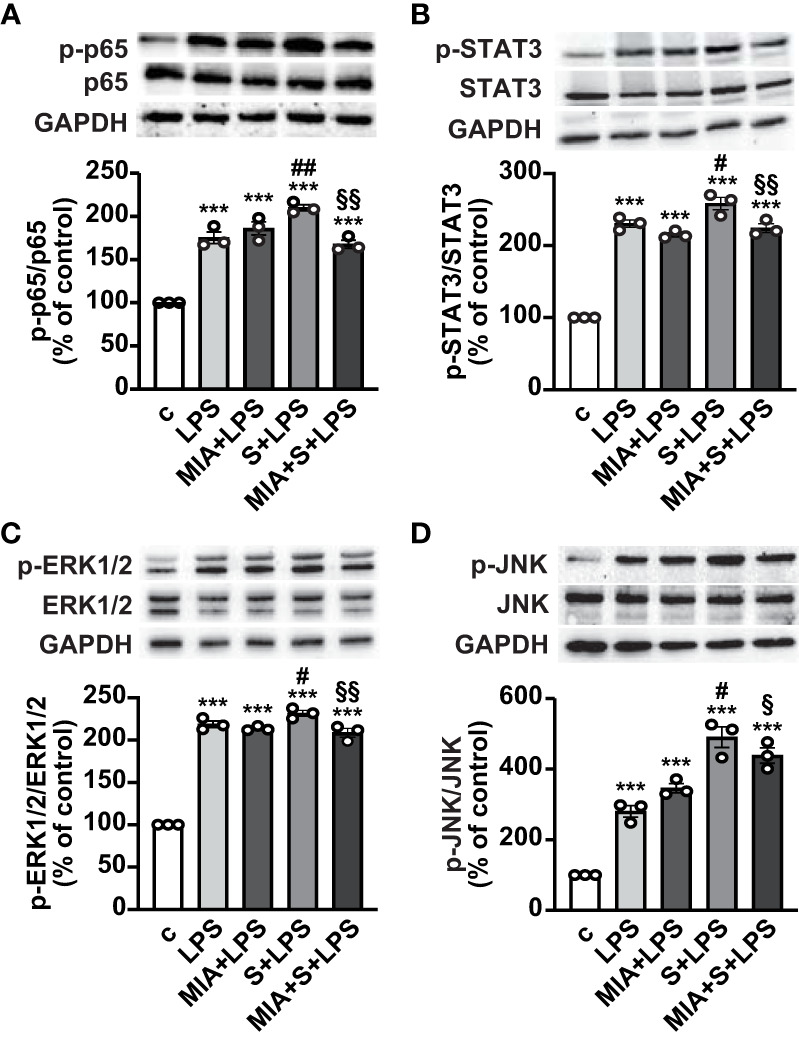
Inhibitory effect of MIA-602 on LPS+S protein-induced activation of inflammatory pathways in THP-1-derived macrophages. Representative Western blots for phosphorylated (p)-p65 **(A)**, p-STAT3 **(B)**, p-ERK1/2 **(C)** and p-JNK **(D)** assessed by Western blot in THP-1 cells either untreated (c, control) or pretreated for 3 h with LPS (50 ng/ml), then up to 24 h with either S protein (500 ng/ml), MIA-602 (1 µM) or both (top panel). Blots were reprobed with non-phosphorylated antibodies for normalization (middle panel) or GAPDH, used as internal control (bottom panel). Graphs show the densitometric analysis of phosphorylated proteins normalized to total proteins and reported as percentage of control (means ± SEM). ***P < 0.001 vs. c; ^#^ *P* < 0.05, ^##^ *P* < 0.01 vs. LPS; ^§^ *P* < 0.05 and ^§§^ *P* < 0.01 vs. LPS+S protein by one-way ANOVA and Tukey’s *post-hoc* test (*n* = 3).

The mechanisms involved in the antinflammatory effects of MIA-602 were next evaluated in THP-1 cells treated with S protein and LPS. Western blot analysis showed that the phosphorylation of the NF-кB subunit p65 was increased by LPS, an effect further potentiated by S protein. MIA-602 had no effect with LPS alone, however, the peptide strongly attenuated the S protein+LPS-induced increase in p65 phosphorylation (**Figure 3A**). Similarly, MIA-602 reduced the phosphorylation of STAT3 (**Figure 3B**), MAPK ERK1/2 and JNK (**Figures 3C, D**), that was enhanced by cotreatment of S protein with LPS, compared with LPS alone. Collectively, these findings indicate that MIA-602 inhibits the inflammatory pathways activated by the combination of S protein and LPS.

### 3.4 MIA-602 reduces inflammatory responses in PBMCs exposed to S protein and LPS combination

**Figure 4:**
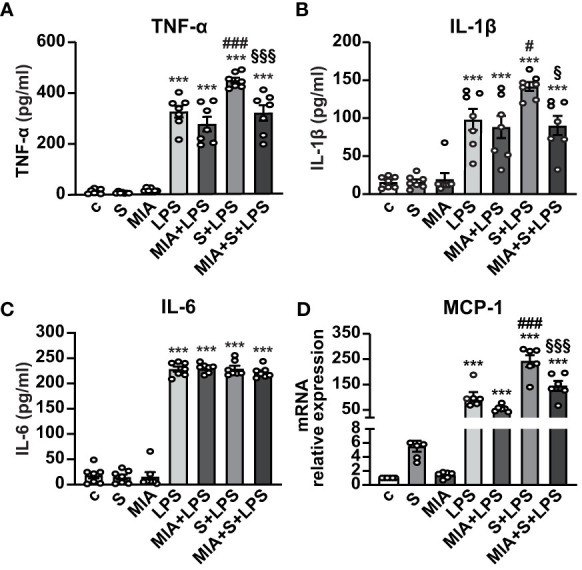
Inhibitory activity of MIA-602 on release and expression of inflammatory cytokines and chemokines in human PBMCs. The cells were either untreated (c, control) or pretreated for 3 h with LPS (10 ng/ml), then up to 24 h with either S protein (500 ng/ml), MIA-602 (1 µM) or both. TNF-α **(A)**, IL-1β **(B)** and IL-6 **(C)** secretion measured by ELISA in cell conditioned medium. **(D)** MCP-1 mRNA expression assessed by real time-PCR. Results are means ± SEM. ***P < 0.001 vs. c; ^#^ *P* < 0.05 and ^###^ *P* < 0.001 vs. LPS; ^§^ *P* < 0.05 and ^§§§^ *P* < 0.001 vs. LPS+S protein by one-way ANOVA and Tukey’s *post-hoc* test (*n* = 7 for **A-C,** *n* = 6 for **D)**.

We next asked whether MIA-602 could inhibit the release of inflammatory cytokines and chemokines in human PBMCs stimulated with S protein and LPS for 24 h. ELISA results showed that S protein alone produced no response, while a robust increase of TNF-α and IL-1β secretion was observed with LPS as single agent, an effect further enhanced by S protein and LPS synergism and counteracted by the addition of MIA-602 (**Figures 4A, B**). By contrast, while LPS alone increased IL-6, the addition of either S protein or the combination of S protein, LPS and MIA-602 showed no effect (**Figure 4C**). As for THP-1 cells, MIA-602 reduced MCP-1 mRNA levels, that were elevated by S protein and LPS combination (**Figure 4D**). These results indicate that, except for IL-6, MIA-602 reduces inflammation in PBMCs stimulated with S protein and LPS.

### 3.5 MIA-602 reduces oxidative stress induced by S protein and LPS combination

**Figure 5:**
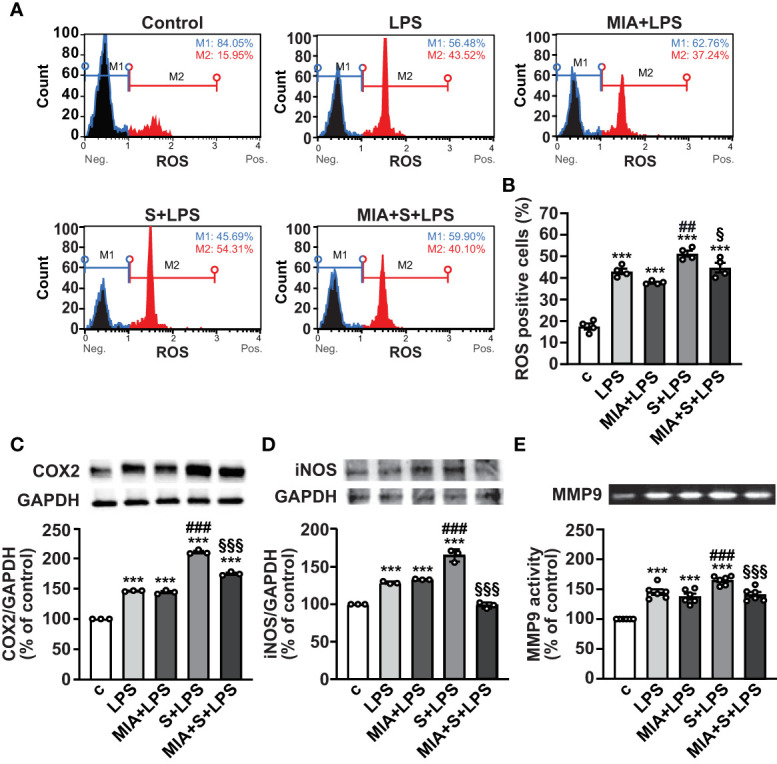
MIA-602-induced protection against oxidative stress in PBMCs. The cells were either untreated (c, control) or pretreated for 3 h with LPS (10 ng/ml), then up to 24 h with either S protein (500 ng/ml), MIA-602 (1 µM) or both. **(A)** Representative images of ROS production assessed by flow cytometry using the Muse^®^ Oxidative Stress Kit. M1: ROS negative cells, M2: ROS-positive cells. **(B)** Graph showing the percentage of ROS positive cells after the different treatments. Results are means ± SEM. ***P < 0.001 vs. control (c); ^##^ *P* < 0.01 vs. LPS; ^§^ *P* < 0.05, vs. LPS+S protein by one-way ANOVA and Tukey’s *post-hoc* test (*n* = 4). COX-2 **(C)** and iNOS **(D)** protein levels assessed by Western blot (top panels). Blots were reprobed with antibody for GAPDH for normalization (bottom panels). **(E)** Representative gelatin zymography of MMP9 activity in PBMC conditioned medium. Graph values show the densitometric analysis of MMP9, reported as percentage of control. Results are means ± SEM. ***P < 0.001 vs. control (c); ^###^ *P* < 0.001 vs. LPS; ^§§§^ *P* < 0.001 vs. LPS+S protein by one-way ANOVA and Tukey’s *post-hoc* test (*n* = 6).

In addition to inflammation, SARS-CoV-2 infection and its complications have been associated with dysregulated redox balance and increased oxidative stress (7, 40). To explore whether MIA-602 reduces oxidative stress induced by S protein and LPS combination, we first examined ROS production in PBMCs by flow cytometry. As expected, LPS alone elevated ROS levels, an effect further enhanced by S protein. MIA-602 was ineffective in cells exposed to LPS alone, however, it reduced ROS production when added to S protein+LPS combination (**Figures 5A**, **B**). Similar findings were obtained for COX-2 and iNOS proteins, whose increase, promoted by S protein and LPS synergism, was blunted by MIA-602 (**Figures 5C, D**).

MMPs have an essential proinflammatory role in lung diseases, and elevated levels of MMP9 have been reported in patients with severe COVID-19, showing positive correlation with the risk of death (41, 42). Gelatin zymography analysis in PBMC conditioned medium showed that the combination of S protein and LPS enhanced the LPS-mediated increase of MMP9, an effect opposed by MIA-602 (**Figure 5E**). Overall, these data indicate that MIA-602 reduces oxidative stress and gelatinase activity induced by S protein and LPS combination in PBMCs.

### 3.6 MIA-602 attenuates NF-кB nuclear translocation and activity in PBMCs stimulated with S protein and LPS combination

**Figure 6:**
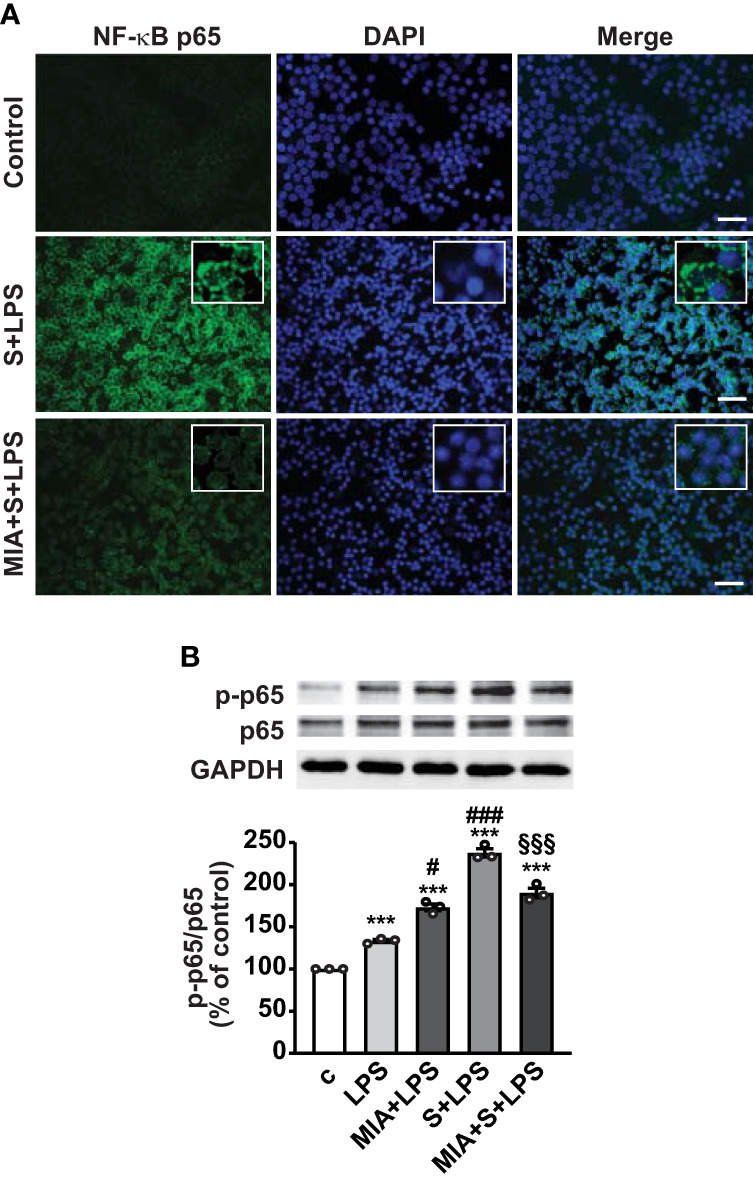
Inhibitory effect of MIA-602 on LPS+S protein-induced translocation and activation of NF-кB in PBMCs. The cells were either untreated (c, control) or pretreated for 3 h with LPS (10 ng/ml), then up to 24 h with S protein (500 ng/ml) or S protein+MIA-602 (1 µM). **(A)** Representative fluorescence microscopy images showing cells stained for NF-кB p65 (green). Nuclei were counterstained with DAPI (blue). Insets show higher magnification (scale bar: 200 μm). **(B)** Representative Western blot for phosphorylated (p)-p65. Blots were reprobed with non-phosphorylated p65 for normalization. Graph shows the densitometric analysis, reported as percentage of control (means ± SEM). ***P < 0.001 vs. c; ^#^ *P* < 0.05, ^###^ *P* < 0.001 vs. LPS, and ^§§§^ *P* < 0.01 vs. LPS+S protein by one-way ANOVA and Tukey’s *post-hoc* test (*n* = 3).

Nuclear translocation of NF-κB is necessary for its activation and transcription of inflammatory mediators (43); thus, we next explored the role of MIA-602 on NF-κB activity in PBMCs. **Figure 6A** shows that nuclear localization of NF-κB subunit p65 was markedly enhanced by S protein and LPS combination, compared with untreated cells, an effect abrogated by MIA-602 (**Figure 6A**). Western blot analysis revealed that MIA-602, although increasing LPS-driven p65 phosphorylation, exerted a strong inhibitory effect when combined with S protein and LPS, compared with S protein+LPS alone (**Figure 6B**). These results indicate that, along with its inhibitory function in THP-1 cells, MIA-602 attenuates NF-κB activity in PBMCs.

## 4 Discussion

The systemic hyperinflammatory response induced by SARS-CoV-2 has been recognized as a major cause of severe COVID-19, with hyperactivation of macrophages and other immune cells to release cytokines and chemokines, which eventually lead to multiorgan failure and ARDS (6). Restraining the uncontrolled release of cytokines is one of the effective ways to counteract the detrimental consequences of COVID-19 (2, 3).

In this work, we show that GHRH antagonist MIA-602 reduces inflammation induced by the combination of SARS-CoV-2 S protein and LPS in THP-1-derived macrophages and human PBMCs. MIA-602 ameliorated the production of cytokines and chemokines, blunted the activation of inflammatory pathways and reduced oxidative stress, main hallmarks of COVID-19.

THP-1 acute monocytic leukemia cell line is commonly used as a substitute for human monocytes, with similar host response and cytokine profile. Additionally, activation with PMA can induce their differentiation into macrophage-like cells, mimicking primary human macrophages in different features (38). However, due to their distinct origin, the two cell models may not completely overlap in terms of response to stimuli (44), which prompted us to also use human PBMCs.

The expression of both GHRH-R and SV1 has been demonstrated in a variety of cells and tissues (45, 46), as well as in normal and diseased human lung (37). Here, we first detected the presence of both pituitary GHRH-R and, although to a lesser extent, of SV1, in THP-1 cells and PBMCs, suggesting that GHRH antagonist may exert its antinflammatory and antioxidant effects by binding to both receptors.

Previous reports have demonstrated the interaction between S protein and LPS, boosting NF-κB activity and inflammatory responses *in vitro*, in THP-1-derived macrophages and PBMCs, and *in vivo* (8–10, 47). Interestingly, patients with metabolic syndrome have high blood levels of bacterial LPS due to gut dysbiosis and are at a higher risk of developing severe COVID-19 (48). Moreover, SARS-CoV-2 infection has been associated with systemic spread of bacteria and microbial products, associated with increased monocyte activation, higher levels of cytokines and chemokines and worse outcomes of the disease, including death (11, 12). These and other data suggest a pathogenetic link between endotoxemia and severe SARS-CoV-2 infection, particularly in patients with comorbidities. Accordingly, we found that the combination of S protein and LPS increased the expression and release of inflammatory cytokines and chemokines and activated inflammatory and oxidative pathways in THP-1 cells and human PBMCs, compared to LPS alone. These effects were counteracted by the addition of MIA-602, which acted as an antinflammatory compound. Interestingly, we could not detect any proinflammatory activity induced by S protein as single agent. This is in line with recent studies demonstrating that S protein-induced cytokine/chemokine production in human primary macrophages is mainly due to endotoxin (i.e., LPS) contamination, while LPS-free recombinant glycosylated S protein has no effect (47, 49). Furthermore, it has been shown that S protein alone, even at high concentrations (10 μg/ml), is inactive on IL-1β secretion in human macrophages from SARS-CoV-2 naïve individuals (50). However, others have demonstrated the ability of S protein alone to trigger inflammation in human and mouse macrophages, although it must be noted that the LPS-induced contamination had not been taken into consideration (51, 52).

The antinflammatory behavior of GHRH antagonists in both cancer and other diseases is well known (19, 28, 30, 32, 35, 36). Indeed, MIA-602, but not GHRH agonist MR-409, reduced the infiltration of macrophages and leucocytes and the production of TNF-α, IL-1β and MCP-1 in a preclinical model of LPS-induced uveitis (31). Similarly, MIA-602, but not MR-409, inhibited LPS-induced elevation of IL-1β, IL-6 and iNOS genes *in vitro*, in ciliary and iris epithelial cells (53). GHRH antagonists also reduced prostate size and expression of inflammatory mediators, such as IL-1β, NF-κB p65, and COX-2, in rat prostatic hyperplasia (54). Here, MIA-602 attenuated the expression and release of TNF-α, IL-1β, IL-8 and MCP-1 but not IL-6, unlike previous findings where MIA-602 was found to decrease TNF-α and IL-6 production *ex vivo in* mouse prefrontal cortex and colon specimens treated with LPS or dextran sulfate sodium (DSS), respectively (34, 55). Interestingly, we also observed that only LPS as single agent was able to elevate IL-6 levels, whereas S protein and LPS synergism produced no further increase, in agreement with a previous study by Petruk et al. (8). It should be also mentioned that, although IL-6 is one of the main cytokines involved in COVID-19 inflammatory response, clinical trials with IL-6 receptor antagonists gave conflicting outcomes, and the effectiveness of IL-6 targeting approach remains to be established (56, 57).

Growing evidence suggests that the endothelium may represent an important therapeutic target in COVID-19. In fact, endothelial dysfunction has been implicated in the pathogenesis and severity of the disease through both direct viral effects and increased inflammatory responses. Moreover, the development of pulmonary disease is associated with excess vascular permeability, hypercoagulation, arterial and venous embolism and endothelial injury, which also contribute to systemic symptoms (58). STAT3 is a master transcription factor of inflammatory genes, involved in the antiviral response in COVID-19 (6). Consistent with our present findings in THP-1 cells, MIA-602 was previously found to suppress STAT3 and MAPK ERK1/2 in bovine pulmonary arterial cells and to support vascular barrier integrity (36). In addition, MIA-602 inhibited the phosphorylation and expression of STAT3 and its downstream inflammatory pathways in LPS-treated human ciliary epithelial cells (30). Interestingly, GHRH agonist JI-34 also attenuated LPS- and pneumolysin (LPY)-induced endothelial dysfunction in human lung microvascular endothelial cells (HL-MVEC), while having no effect on inflammation (59), suggesting that GHRH agonists and antagonists may elicit similar protective effects in specific settings. However, it has been demonstrated that the antinflammatory and antioxidative activities of MIA-690 *in vivo* are greater than those of the GHRH agonist MR-409, likely due to inhibition of IGF-I or stronger direct effects of the antagonists (55).

Of note, a recent study showed that adult individuals with untreated isolated GH deficiency (IGHD) due to a GHRH-R gene mutation, characterized by severe short stature, extremely low GH and undetectable IGF-I levels, cope better with SARS-CoV-2 infection than those with normal GH (60). Additionally, macrophages from individuals with the same type of IGHD were found to be less prone to Leishmania infection compared to those of normal subjects (61). The authors of these studies suggested that the immunological benefits could be related to IGF-I deficiency, resulting in reduced cell replication, lower DNA damage, as well as decreased IGF binding protein, and insulin. In line with the above studies, we suggest that MIA-602 may reduce inflammation in THP-1-derived macrophages and PBMCs in part by attenuating the autocrine-paracrine activities of locally produced IGF-I and related inflammatory pathways (19, 62).

Here, MIA-602, besides decreasing cytokine levels, also blunted NF-κB activity, which is implicated in the pathogenesis of severe COVID-19 (63). Activation of NF-κB by inducers such as bacterial LPS, ROS, cytokines and viral DNA and RNA, promotes the expression of a wide range of cytokines, chemokines, and oxidative stress components like iNOS and COX-2 (3, 41). Accordingly, our results showed the ability of MIA-602 to also reduce oxidative stress, by attenuating ROS production, as well as iNOS and COX-2 protein levels in PBMCs exposed to S protein and LPS synergism. This is consistent with the antioxidative effects of GHRH antagonists, including MIA-602, demonstrated in different cell types and models of inflammation and cancer (24, 25, 30, 32, 34, 54, 55, 64). Interestingly, enhanced ROS production has been linked to reduced antiviral host responses and increased virus-induced inflammation and apoptosis, with activation of MAPK pathway and elevation of inflammatory cytokines (7). Our results also demonstrate that MIA-602 reduces MMP9 activity in PBMCs exposed to S protein and LPS combination, in agreement with the previously observed inhibitory effect of the peptide in human pleural mesothelioma cell lines, inflamed prostate epithelial cells and LPS-stimulated ciliary epithelial cells (24, 25, 30, 32). Noteworthy, beyond being a key mediator of tumor growth, metastasis and angiogenesis, by degrading extracellular matrix components, MMP9 has an important role in lung physiopathology and inflammatory responses (65), and its elevation has been associated with increased risk of death in patients with severe COVID-19 (41, 42).

In conclusion, in addition to corroborating the previously described antinflammatory and antioxidative functions, our findings indicate that the effects of GHRH antagonists could be extended beyond conditions such as cancer and inflammation. In fact, to the best of our knowledge, the novelty of this study consists in the fact that: i) MIA-602 acts in macrophage-like cells and PBMCs, key players in the immune system and inflammatory responses, ii) MIA-602 attenuates inflammation and oxidative stress induced by the combination of a viral protein with a bacterial component, and iii) MIA-602 inhibits signaling pathways involved in the pathogenesis and progression of severe COVID-19. Overall, our results suggest that the development of GHRH antagonists may lead to new therapies against COVID-19 and related comorbidities, including lung diseases and sepsis-induced ARDS.

## Data availability statement

The raw data supporting the conclusions of this article will be made available by the authors, without undue reservation.

## Ethics statement

Experiments using human PBMCs were conducted according to the guidelines of the Declaration of Helsinki and approved by the Ethic Committee of A.O.U. Città della Salute e della Scienza di Torino, Turin, Italy (CS21255 - Protocol #0050416, May 16, 2019). Participants provided written informed consent to participate in this study. 

## Author contributions

RG and GG contributed to conception and design of the study. GG, IG, SB, FP, RK, LB, DB and TL performed the experiments and analyzed the data. RC, WS and AS provided MIA-602. MB, EG and AS revised the paper. RG supervised the work and wrote the final manuscript. All authors contributed to the revision of the manuscript and approved the submitted version.
